# A Tailored Web-Based Intervention to Improve Parenting Risk and Protective Factors for Adolescent Depression and Anxiety Problems: Postintervention Findings From a Randomized Controlled Trial

**DOI:** 10.2196/jmir.9139

**Published:** 2018-01-19

**Authors:** Marie Bee Hui Yap, Shireen Mahtani, Ronald M Rapee, Claire Nicolas, Katherine A Lawrence, Andrew Mackinnon, Anthony F Jorm

**Affiliations:** ^1^ Monash Institute of Cognitive and Clinical Neurosciences School of Psychological Sciences Monash University Clayton Australia; ^2^ Melbourne School of Population and Global Health University of Melbourne Melbourne Australia; ^3^ Centre for Emotional Health Macquarie University New South Wales Australia; ^4^ Black Dog Institute University of New South Wales Sydney, New South Wales Australia

**Keywords:** family, anxiety, parenting, depression, adolescent, Internet, mental health, preventive health services

## Abstract

**Background:**

Depression and anxiety disorders in young people are a global health concern. Parents have an important role in reducing the risk of these disorders, but cost-effective, evidence-based interventions for parents that can be widely disseminated are lacking.

**Objective:**

This study aimed to examine the postintervention effects of the Partners in Parenting (PiP) program on parenting risk and protective factors for adolescent depression and anxiety, and on adolescent depression and anxiety symptoms.

**Methods:**

A two-arm randomized controlled trial was conducted with 359 parent-adolescent dyads, recruited primarily through schools across Australia. Parents and adolescents were assessed at baseline and 3 months later (postintervention). Parents in the intervention condition received PiP, a tailored Web-based parenting intervention designed following Persuasive Systems Design (PSD) principles to target parenting factors associated with adolescents’ risk for depression and anxiety problems. PiP comprises a tailored feedback report highlighting each parent’s strengths and areas for improvement, followed by a set of interactive modules (up to nine) that is specifically recommended for the parent based on individually identified areas for improvement. Parents in the active-control condition received a standardized package of five Web-based factsheets about adolescent development and well-being. Parents in both conditions received a 5-min weekly call to encourage progress through their allocated program to completion. Both programs were delivered weekly via the trial website. The primary outcome measure at postintervention was parent-reported changes in parenting risk and protective factors, which were measured using the Parenting to Reduce Adolescent Depression and Anxiety Scale (PRADAS). Secondary outcome measures were the adolescent-report PRADAS, the parent- and child-report Short Mood and Feelings Questionnaire (depressive symptoms), and parent- and child-report Spence Children’s Anxiety Scale (anxiety symptoms).

**Results:**

Parents in the intervention condition completed a mean of 73.7% of their intended personalized PiP program. A total of 318 parents (88.6%, 318/359) and 308 adolescents (92.8%, 308/332) completed the postintervention assessment. Attrition was handled using mixed model of repeated measures analysis of variance. As hypothesized, we found a significant condition-by-time interaction on the PRADAS, with a medium effect size, Cohen *d*=0.57, 95% CI 0.34-0.79. No significant differences between conditions were found at postintervention on any of the secondary outcome measures, with adolescent depressive (parent-report only) and anxiety (both parent- and adolescent-report) symptoms decreasing significantly from baseline to postintervention in both conditions.

**Conclusions:**

The fully automated PiP intervention showed promising short-term effects on parenting behaviors that are associated with adolescents’ risk for depression and anxiety. Long-term follow-up is required to ascertain whether these effects translate into reduced adolescent depression and anxiety problems. The intervention may be useful as a low-cost universal public health program to increase parenting practices believed to benefit adolescents’ mental health.

**Trial Registration:**

Australia New Zealand Clinical Trials Registry: ACTRN12615000328572; https://www.anzctr.org.au/ Trial/Registration/TrialReview.aspx? id=368274 (Archived by WebCite at http://www.webcitation.org/6qgsZ3Aqj)

## Introduction

### The Problem of Depression and Anxiety Disorders in Youth

Depression and anxiety disorders are among the most common mental disorders affecting 18% and 38%, respectively, of young people in the age range of 13 to 17 years [1]. Developing these disorders early in life, especially if left untreated, can increase young people’s suicide risk and forecast a wide range of psychosocial and vocational impairments, resulting in deleterious long-term sequelae [2-4]. Although intervention efforts for these disorders continue to progress, a large proportion of the burden of disease is still unavertable even with optimal treatment [5]. There is, hence, an urgent need for an effective, integrated approach to prevent these disorders. As the incidence of depression and anxiety disorders peaks during adolescence, early adolescence is a particularly opportune time to target preventive efforts [6]. Fortunately, evidence to date indicates that depression and anxiety disorders in young people can be prevented [7-9].

### Parents Have an Important Role in Prevention

There is now substantial robust evidence delineating risk and protective factors for adolescent anxiety and depressive disorders [10,11]. Importantly, some of these factors are within parents’ control or influence and are potentially modifiable [12]. Synthesizing longitudinal, retrospective, and cross-sectional evidence, a recent review identified a sound evidence base for three protective parental factors for depression (warmth, autonomy granting, and monitoring) and one for anxiety (warmth). Three risk factors for both outcomes were also identified: interparental conflict, overinvolvement, and aversiveness [13]. However, despite this evidence base, parents’ knowledge about their role in reducing their adolescent’s risk of depression is less than optimal [14], highlighting a need to equip parents in the general population with evidence-based preventive resources.

### Existing Preventive Parenting Interventions

Preventive parenting programs have been developed to capitalize on parents’ influence on their child’s development and mental health based on the underlying assumption that changing parenting (mediators) will in turn change a child’s risk for depression and anxiety [12]. A recent systematic review and meta-analysis found that preventive interventions primarily targeting parents (ie, most of the intervention is with the parent, as opposed to the child or involving the whole family) have beneficial effects on the child’s internalizing (depression and anxiety) outcomes lasting up to 11 years post intervention [9]. This contrasts with the evidence for preventive interventions targeting young people directly, which have observed intervention effects lasting less than 2 years [15,16]. Although this evidence base highlights the remarkable promise of preventive parenting interventions, only 3 of the 51 parenting interventions included in the review were designed for parents of adolescents [9]. Moreover, many parenting programs are not well used even when available because of barriers such as scheduling difficulties and privacy concerns [17].

Preventive parenting interventions can be *universal* (ie, delivered to all parents regardless of risk), *selective* (targeting parents whose children have known risk factors), or *indicated* (targeting parents whose children show signs or symptoms of emerging disorders) [18]. Universal programs tend to have a smaller effect than selective or indicated programs at the level of the individual [16]. However, they can have a great public health impact because they reach a larger proportion of the population [19] and have the potential to shift the population mean levels of depression and anxiety symptoms [19]. Notably, in the aforementioned review of preventive parenting interventions [9], there was no evidence that type of prevention (universal, selective, or indicated) moderated intervention effects. As highlighted in the Institute of Medicine report [18], universal interventions are advantageous when they are effective and acceptable, have a low cost per individual, and carry a low risk of harm. When trying to engage parents in prevention of mental health problems in their child, universal approaches can increase acceptability because they minimize the perceived stigma that some parents fear would be attached to themselves as “bad” parents, or to their child as having problems needing intervention [20]. Hence, a universal program should be considered an integral component in a public health approach to empower parents for their role in prevention of youth depression and anxiety disorders [9].

### Potential of a Web-Based Parenting Intervention

Web-based media are a promising mode of delivering universal prevention programs because of their scalability and likely cost-effectiveness [18]. Universal programs are also well-suited to Web-based delivery because they usually involve a lower intensity of intervention (eg, require little or no contact with trained professionals), hence, reducing the cost of population-wide dissemination. Given the increasing reach of the Internet [21], Web-based media have been recommended as one effective way to increase participation in preventive interventions [22]. Web-based universal parenting programs also have the potential to overcome the aforementioned barriers of existing face-to-face programs because of their anonymity, flexibility, and accessibility. The Internet has become a popular source of information on parenting and child mental health among parents [2,23], and a recent survey found that the idea of a tailored online parenting program for parents of adolescents was viewed favorably [24]. Moreover, implementation fidelity is guaranteed by the computerized delivery of a well-designed and well-maintained program [25]. Despite these potential benefits, a recent systematic review [9] failed to identify any evidence-based, tailored Web-based parenting intervention designed to prevent adolescent depression and anxiety disorders. The potential of online prevention programs targeting parents of adolescents remains as yet, largely untapped [26], but these programs would comprise a promising public health approach to preventing adolescent depression and anxiety that is potentially lower in cost per individual compared with existing programs.

The Partners in Parenting (PiP) program is a tailored Web-based parenting intervention to prevent adolescent depression and anxiety problems (see [27] for more details). Its content is derived from Parenting Guidelines [28], which were developed through a rigorous two-stage process involving a systematic review of parenting risk and protective factors associated with adolescent depression and anxiety [12]; and a Delphi study of international expert consensus about parenting strategies that are important for reducing risk for adolescent depression and anxiety disorders [29]. The program development process was aligned with a consumer-engagement approach [30] by involving parents of adolescents in reference group workshops and obtaining input from adolescents through focus group consultations. Design of the Web-based components of PiP were guided by the Persuasive Systems Design (PSD) model that proposes to purposefully use technology to influence behavior change [31] and has been found to influence adherence to Web-based interventions [32]. For example, following the PSD tailoring principle, the program’s automated tailoring feature screens each parent on a wide range of parenting factors known to influence risk for adolescent depression and anxiety. This identifies areas for improvement to target in each parent’s personalized intervention. This tailoring feature increases the perceived relevance of the program for each parent [33], and potentially its effects [32], without requiring the costly involvement of trained professionals, hence increasing potential for scalability and sustainability [33]. As a preventive parenting intervention, PiP is designed to increase parental protective factors and decrease parental risk factors associated with adolescent depression and anxiety. The change in parenting factors (proximal outcome and direct target of the intervention) is in turn expected to reduce adolescent risk for depression and anxiety problems in the long term.

The primary aim of this study was to evaluate the effects of PiP compared with an active-control condition (educational factsheets on adolescent development and well-being) in a randomized controlled trial (RCT). Specifically, we hypothesized that compared with the control group, parents who received PiP will show (1) greater improvements in parenting risk and protective factors from baseline to postintervention (primary outcome) using the Parenting to Reduce Adolescent Depression and Anxiety Scale (PRADAS; [34]), a criterion-referenced measure of parenting against the Parenting Guidelines [28], and (2) greater reductions in adolescent depression and anxiety symptoms and greater improvements in adolescent-report parenting from baseline to postintervention (secondary outcomes).

Although the ultimate aim of the PiP is the prevention of adolescent depression and anxiety problems, this parenting intervention is posited to result in adolescent benefits indirectly through its effects on parenting. The RCT includes a postintervention assessment (focus of this paper), where the primary outcome of interest is the intervention’s proposed mechanism of change: parenting risk and protective factors. Adolescent depressive and anxiety symptoms are secondary outcomes at postintervention because we expect the intervention’s effects on these outcomes to take time, operating through changes in parenting. However, beyond the scope of this paper, the RCT also includes a 12-month follow-up assessment, which is currently being undertaken, when the primary outcomes of interest will be adolescent depressive and anxiety symptoms.

## Methods

### Trial Design

This study was a parallel-group superiority RCT with parent-adolescent dyads randomly allocated in a 1:1 ratio to one of the two conditions: (1) PiP or (2) educational factsheets (control intervention). The trial was prospectively registered with the Australian New Zealand Clinical Trials Registry (ACTRN12615000328572; see Multimedia Appendix 1 for Consolidated Standards of Reporting Trials [CONSORT-EHEALTH checklist]).

### Setting, Participants, and Eligibility Criteria

From August 2015 to September 2016, 359 parent-adolescent dyads were recruited primarily via government, Catholic, and independent schools across the state of Victoria in Australia. Schools were contacted by email and phone to request that recruitment flyers (hard copy or electronic) were distributed to parents of students in Years 7 to 10 (aged 12-15 years). Other means of recruitment included disseminating advertisement flyers via social media, online networks, and through mental health organizations (eg, *beyondblue* and Mental Health First Aid Australia). Interested and eligible parents were invited to register via the dedicated trial website. To be eligible, parents had to have a target child in the age range of 12 to 15 years, regular access to the Internet and an email account, and reside in Australia. Computer or Internet literacy was an implicit eligibility criterion. Parents were asked to provide consent and contact details for their child to participate in the trial but could still participate if their adolescent declined participation. For each family, only one parent and one child could be included in the trial (see Multimedia Appendix 2 for participant informed consent documentation). Participants were not excluded if the adolescent scored in the clinically elevated range (as determined by published clinical cut-off scores) on either the depressive or anxiety symptom measures (either parent- or child-report) at baseline.

**Figure 1:**
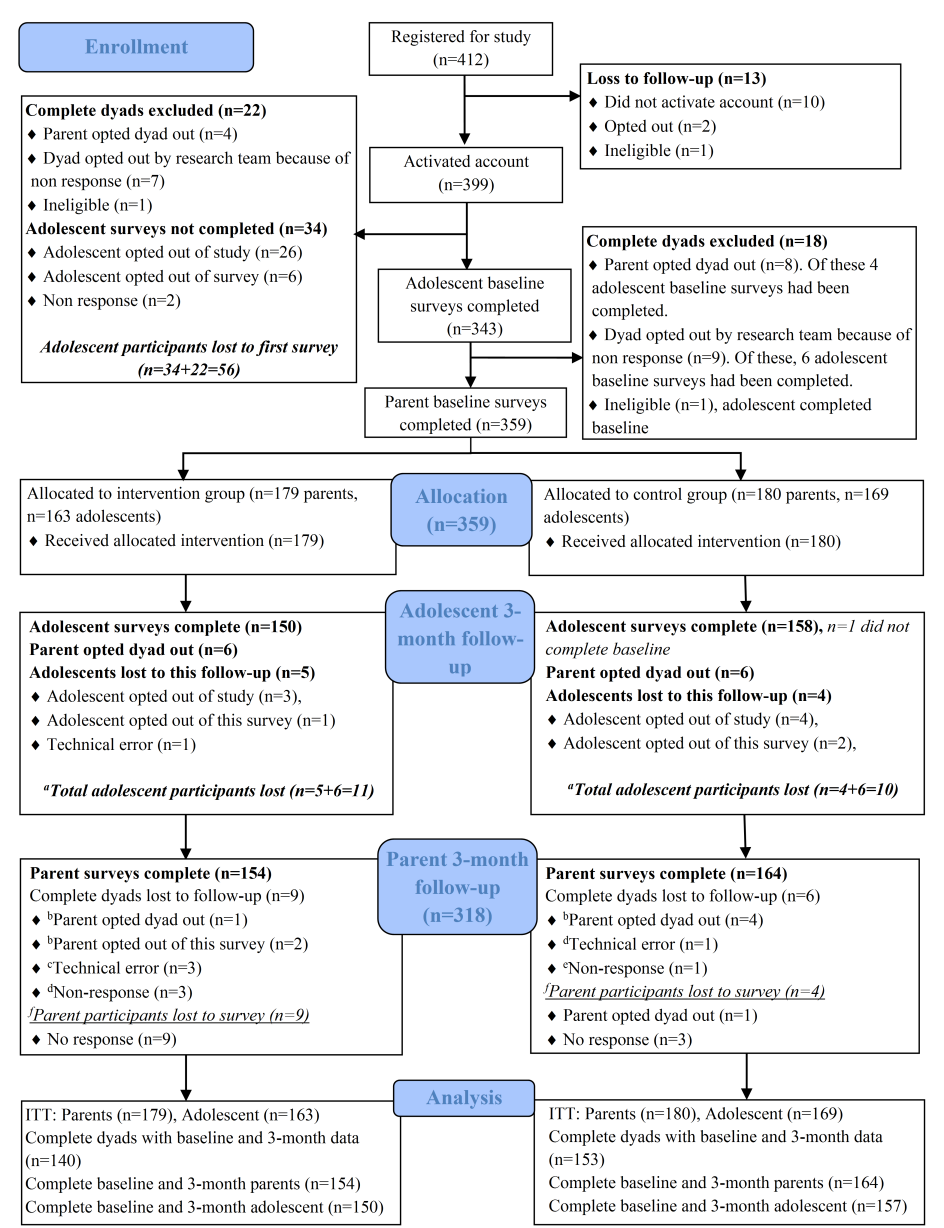
Participant flow diagram. ITT=Intention-to-treat analyses. Parent or dyad remain enrolled in study unless indicated that they had opted out. aIncludes complete dyads opted out; badolescent opted out of study at 3-month follow-up; cof these, 2 adolescent participants had opted out before completing adolescent baseline survey and, one adolescent’s 3-month follow-up was also missed because of a technical error; dadolescent participant(s) opted out before completing adolescent baseline survey; eadolescent participant opted out of completing 3-month follow-up survey; and fadolescent participant completed 3-month follow-up.

Figure 1 shows the study design and flow of participants. The study was primarily conducted online via automated emails to parents and a dedicated RCT website through which parents received their allocated intervention and parent and adolescent participants completed their study assessments. Adolescent assessments were completed online with the assistance of a research officer over the phone. Parents received an automated email inviting them to complete their online assessment as soon as their child had submitted their corresponding assessment responses or declined to participate.

### Interventions

In addition to receiving their Web-based program (described below), all parent participants received a weekly phone call from a researcher, starting 7 days after completing their baseline survey and every week thereafter until they had completed their allocated intervention. In the intervention group, the total number of weekly calls was designed to match the number of modules in each parent’s tailored parenting program. However, if they had less than five modules, they still received five calls (to match the number of calls received by the control group). Research staff were trained to make these calls following a standard protocol (ie, a flowchart of prompts and appropriate responses, eg, “Did you [complete your module or read your factsheet] this week?” and “Did you try to put into practice or apply any of the information you read?”) and did not provide individual advice or therapy. These calls were intended to address any study-related questions or troubleshoot technical issues that arose, encourage parents to progress through their allocated intervention each week till completion, and enhance parents’ engagement.

#### The Partners in Parenting Intervention

PiP [27] is a Web-based parenting program that is part of the broader Parenting Strategies research translation online platform [35]. The programming of the intervention was first completed in July 2015 and was not modified throughout the trial.

On the basis of their responses to a self-assessment parenting scale (the PRADAS [34]), parents in the intervention condition received an individually tailored feedback report that highlighted areas where they were doing well (ie, concordant with the Parenting Guidelines [28]) and areas where they could improve (ie, not concordant with the Guidelines). They were then given access to the Web-based modules (up to nine) to support them in making changes to identified areas for improvement [27]. Specific modules were recommended to parents based on their responses to the PRADAS at baseline. Table 1 shows the alignment of topics across the Guidelines, PRADAS, feedback report, and modules (screenshots are available in Multimedia Appendix 3).

Feedback messages in PiP are brief, with practical strategies provided in dot point form and are designed to motivate behavior change [27]. Parents viewed their feedback report on the website immediately after submitting their online baseline assessment and being randomly allocated to the intervention condition. They were also emailed a copy of their feedback report, the Parenting Guidelines, and instructions on accessing the interactive Web-based parenting program with their recommended modules.

Upon logging in to their parenting program, parents were presented with their recommended modules, as well as other available modules. They could further tailor their program at this stage by deselecting recommended modules and/or selecting additional modules. They then confirmed their selection and commenced their personalized program. The nine modules comprising the PiP intervention were derived from topics covered in the Parenting Guidelines (see Table 1). Modules include illustrations, audio clips, vignettes, interactive activities, goal-setting exercises, and an end-of-module quiz with immediate feedback to consolidate learning. Each module is designed to help parents make changes to their parenting so as to become more concordant with the Guidelines. Each module takes about 15 to 25 min to complete. Parents were directed to their first selected module immediately after they had completed their baseline assessment and received their feedback report. Thereafter, parents were notified via weekly automated emails as their next module was made available for them through their personalized dashboard until they had completed their whole program. One module is made available for parents every 7 days, in a set order, regardless of whether they had completed preceding modules. After completing their program, parents had unlimited access to all PiP modules for the duration of the RCT (up to 3 years for parents who registered early in the recruitment phase of the 3-year RCT, ie, August 2015).

#### Educational Factsheets (Control Intervention)

Parents in the control condition were provided with a standardized package of educational materials about adolescent development and mental health via the trial website. Each week for 5 weeks, parents received an automated email inviting them to access their factsheet for that week (to match the expected mean number of modules received by the intervention group). To mirror the experience of intervention group parents who accessed each module on the trial website, control group parents accessed each factsheet by logging in to their dashboard on the website. The factsheets provide general information to parents as opposed to tailored, actionable strategies and were designed to represent a selection of resources that are available to parents as part of the current health promotion approach for adolescent well-being. The materials were adapted from credible existing resources provided on the Raising Children Network website [36]. The topics of the five factsheets were (1) Teen development: an overview, (2) The teenager’s developing brain, (3) The teenager’s changing body, (4) Resilience, and (5) Happy teenagers and teenage well-being. Parents had access to these factsheets for the duration of the RCT.

**Table 1:** Guidelines topics, corresponding sections of the parenting scale (Parenting to Reduce Adolescent Depression and Anxiety Scale, PRADAS) and personalized feedback report, title of interactive modules, and outline of content.

Guidelines subheading	Corresponding section of the PRADAS^a^ and feedback report	Title of interactive module	Outline of content
You can reduce your child’s risk of developing depression and clinical anxiety	Not applicable (NA). Not included in the PRADAS or feedback report	NA. No module on this topic	Psychoeducation about the role of parents in the prevention of adolescent depression and anxiety
Establish and maintain a good relationship with your teenager	Your relationship with your teenager	Connect	Acknowledges the challenge of connecting with adolescent children and provides specific tips on how to do this
Be involved and support increasing autonomy	Your involvement in your teenager’s life	Nurture roots and inspire wings	Helps parents establish the important balance between staying involved and interested in their adolescent’s life, while encouraging increasing age-appropriate autonomy
Encourage supportive relationships	Your teenager’s relationships with others	Good friends, supportive relationships	Provides strategies for parents to support their adolescent’s social skills development
Establish family rules and consequences	Your family rules	Raising good kids into great adults: establishing family rules	Highlights the importance of consistent and clear boundaries for adolescent behaviors and provides specific strategies to establish these
Minimize conflict in the home	Your home environment	Calm versus conflict	Addresses the need for adaptive conflict management between parents and between parent and adolescent and provides specific strategies to do these
Encourage good health habits	Health habits	Good health habits for good mental health	Provides strategies to help parents encourage good health habits in their adolescent, including a healthy diet, physical activity, good sleep habits, and abstinence from alcohol and drugs
Help your teenager to deal with problems	Dealing with problems in your teenager’s life	Partners in problem solving	Provides strategies for parents to help their adolescent develop good problem-solving and stress management skills
Help your teenager to deal with anxiety	Coping with anxiety	From surviving to thriving: helping your teenager deal with anxiety	Provides strategies for parents to help their adolescent manage their everyday anxiety
Encourage professional help seeking when needed	Getting help when needed	When things aren’t okay: getting professional help	Helps parents understand what depression and anxiety problems can look like in adolescents, and what they can do if their adolescent is or becomes unwell
Don’t blame yourself	Don’t blame yourself (included for all parents in feedback report only)	NA. No module on this topic	Aims to dispel guilt or self-blame in parents

^a^PRADAS: Parenting to Reduce Adolescent Depression and Anxiety Scale.

### Outcomes

Parents and adolescents were asked to complete online assessments comprising the following measures at baseline and 3 months later. The latter time point was designated as the postintervention assessment based on the expectation that most parents would have completed their intervention (maximum of nine modules, one per week) by then.

#### Primary Outcome Measure: Parenting to Reduce Adolescent Depression and Anxiety Scale (PRADAS)

The PRADAS [34] is a criterion-referenced measure assessing parents’ current parenting behaviors against specific recommendations in the Parenting Guidelines (the “criterion”; [28]). Newly developed for use in this study, the original version consisted of 79 questions assessing the nine domains of parenting addressed in the Parenting Guidelines. Following validation analyses [34], the scale was revised to include 73 of the original 79 items after dropping one 6-item subscale (“Your teenager’s relationships with others”). The final scale assesses each parent’s total concordance with the Parenting Guidelines across eight domains of parenting assessed using eight subscales (8-12 items per subscale). Each item corresponds to a parenting recommendation in the Guidelines and involves either a 4-point Likert-type frequency scale (for specific parenting behaviors such as showing affection to their child; eg, never, rarely, sometimes, or often) or a likelihood scale (for hypothetical scenarios such as noticing a persistent change in their adolescent’s behavior; eg, very unlikely, unlikely, likely, or very likely). See Multimedia Appendix 3 for sample items.

For each item, certain responses were prespecified by the authors [34] as indicating concordance with the Guidelines (scored as 1), with the remaining responses for the item deemed “nonconcordant” (scored as 0). Scores for all 73 items were summed to yield the total concordance score, which can range from 0 to 73. The PRADAS total score demonstrated good reliability (including coefficient of agreement and test-retest reliability) and convergent validity in an Australian validation sample comprising 711 parents, which included data from the current RCT [34]. Test-retest reliability for the PRADAS total score was *r*=.76, *P*<.001 for a smaller subsample of 175 parents based on data collected 1 month apart [34]. The coefficient of agreement for the PRADAS total score in the current sample was 0.97 at baseline and 0.96 at 3-month follow-up assessment.

#### Secondary Outcome Measure #1: Short Mood and Feelings Questionnaire (SMFQ)

The Short Moods and Feelings Questionnaire (SMFQ) is the brief 13-item version of the Mood and Feelings Questionnaire [37], which assesses depressive symptoms in children and adolescents using child-report (SMFQ-C) or parent-report (SMFQ-P). Respondents indicate the frequency of depressive symptoms in the past 2 weeks by rating each item on a 3-point scale, with 0=not true, 1=sometimes true, and 2=true. Both parents and adolescents reported on adolescent depressive symptoms in this study; Cronbach alphas were .91 and .90, respectively, at baseline and .91 and .92 at postintervention.

#### Secondary Outcome Measure #2: Spence Children’s Anxiety Scale (SCAS)

Adolescent anxiety symptoms were assessed using the *Spence Children’s Anxiety Scale-Child version* (SCAS-C) [38] and SCAS for parents (SCAS-P; [39]). Both versions have 39 items assessing six domains of anxiety in children: separation anxiety, social phobia, obsessive-compulsive disorder, panic disorder or agoraphobia, generalized anxiety, and fear of physical injury. Respondents rate the frequency of anxiety symptoms on a 4-point scale, from 0=never to 3=always. Item responses are summed to form a total anxiety score, which has demonstrated acceptable-to-high reliability in children in the age range of 8 to 14 years. Cronbach alphas were .91 and .92 at baseline and .94 and .95 at postintervention for parent and adolescent versions, respectively.

#### Secondary Outcome Measure #3: Parenting to Reduce Adolescent Depression and Anxiety Scale-Adolescent (PRADAS-A) Report

An adolescent-report version of the PRADAS, the PRADAS-A, was developed and validated by the research team, also as a criterion-referenced measure (Cardamone-Breen, unpublished data, 2017). The original PRADAS-A has 47 items and assesses a subset of parenting behaviors across the same nine domains assessed in the PRADAS. Following validation analyses, the same “Relationships with others” subscale (four items) was dropped. The final PRADAS-A comprises 43 items in eight subscales (2-7 items per subscale). Most items involve a 4-point Likert-type frequency response scale (ie, never, rarely, sometimes, and often) except for the last 2-item subscale “Getting help when needed,” which utilized a 4-point likelihood scale (ie, very unlikely, unlikely, likely, and very likely).

Scoring of responses on each item as reflecting parenting behaviors that are concordant (scored as 1) or nonconcordant (scored as 0) with the Parenting Guidelines was predetermined by the scale authors. Scores for all 43 items were added to form the total concordance score, which can range from 0 to 43. The PRADAS-A total score demonstrated good reliability (including coefficient of agreement and test-retest reliability) and convergent validity in an Australian validation sample comprising 670 adolescents in the age range of 12 to 15 years, which included data from the current trial (Cardamone-Breen, unpublished data, 2017). Test-retest reliability for the PRADAS-A total score was r=.81, *P*<.001 for a smaller subsample of 160 adolescents participants based on data collected 3 months apart (Cardamone-Breen, unpublished data, 2017). The coefficient of agreement for the PRADAS-A total score in the current sample was 0.97 at both baseline and 3-month follow-up assessments.

### Intervention Completion and Adherence

We defined intervention adherence following Kelders and colleagues [40] whereby percentage of individuals who adhere to the intervention=100% x [(number of participants whose observed usage equals their intended usage of the Web-based intervention)/(total number of individuals who received the intervention)]. We operationalized intervention completion as percentage of program completed=100% x [(observed usage of the Web-based intervention) or (intended usage of the Web-based intervention)]. For the PiP condition, intended usage is the total number of modules the parent had locked in to their personalized program after reviewing the program’s recommendations and applying their personal preferences. Observed usage is defined as the total number of modules in their personalized program that were completed. A module was considered to be completed if it had a “closed” timestamp, there were responses recorded for its end-of-module quiz, or the selected goal for the module had been checked off as achieved by the parent. A closed timestamp for each module was stored in the database when the parent clicked on “Finish module” on the last page of the module. For the control condition, intended usage is fixed as five factsheets, whereas observed usage was the total number of factsheets with a closed timestamp. A closed timestamp for each factsheet was saved as long as the factsheet was clicked on and “opened” on the website.

### Sample Size

For a repeated measures design, with one preintervention measure and two postintervention measures, using the analysis of covariance method of sample size calculation and assuming a 0.70 correlation between pre-post measurements, to detect a small effect size (Cohen *d*=0.20), with a power of 0.80 and a Cronbach alpha of .05, we required a total sample of 294 parent-adolescent dyads (147 dyads per group). Allowing for up to 15% attrition, we aimed to recruit 338 parent-adolescent dyads (169 dyads per group).

### Randomization

The random sequence generation was automated within the trial website via software architecture, with participant assignment revealed to parents only after parent and adolescent (if applicable) baseline assessments had been completed, hence, ensuring allocation concealment. Parent-adolescent dyads were randomly assigned in a 1:1 ratio with no stratification, resulting in 179 dyads allocated to PiP and 180 dyads to the control condition.

### Blinding

It was not possible to blind parents to their assignment because of the informed consent procedures. The research officers who spoke to parents for their weekly calls were also not blinded to the parent’s assignment as they had to tailor the script to match the program the parent was receiving. Adolescents were not informed of their parent’s assignment, so were assumed to be “blinded” to this.

### Statistical Methods

All statistical analyses were completed with Statistical Package for the Social Sciences (SPSS) version 24.0 (IBM Corp) software. We conducted independent *t* tests and chi-square tests to test for differences between conditions at baseline in outcome measures and demographic data. We also compared the baseline characteristics of those who completed postintervention assessments and those who did not, to explore possible attrition biases.

We conducted all analyses on an intention-to-treat basis, using mixed model of repeated measures (MMRM), a likelihood-based approach that utilizes all available data, including those from participants who withdrew from the trial after completing the baseline assessment. MMRM produces unbiased estimates of intervention effects under the assumption that data are missing completely at random or missing at random [41].

For MMRM, intervention group (condition) and time ie, assessment wave) and the interaction between condition and time were set as fixed factors.

Post-hoc analyses were also conducted to explore whether intervention effects differed depending on adolescents’ depression or anxiety symptom levels at baseline. SPSS PROCESS macros [42] were used to conduct these moderation analyses, with the predictor of condition coded as 1=PiP and 0=control, mean-centered baseline SCAS or SMFQ score as the continuous moderator, and change in outcome variables computed by subtracting baseline scores from postintervention scores. To minimize shared method variance, we relied on different informants (parent vs adolescent) for the moderator versus outcome variables. For example, to assess effects on SMFQ-P, we used SMFQ-C scores as the moderator. To minimize the number of post-hoc analyses conducted, when examining a parenting outcome according to one informant, we relied on the other informant for the moderator (one of the two symptom measures). Hence, for example, the PRADAS was the outcome variable in two moderation analyses, one using change in SMFQ-C scores as the moderator and the other using the SCAS-C.

All tests were conducted using Cronbach alpha level of .05 and 95% CIs.

### Ethics and Informed Consent

This RCT was approved by the Monash University Human Research Ethics Committee, CF14/3887-2014002024. Informed consent was obtained from parent participants at registration via checkboxes on the trial website, and verbal assent from adolescent participants was obtained over the phone.

### Safety Protocols

Participants were followed up by a provisional psychologist (postgraduate doctoral candidate in clinical psychology) if the parent-adolescent dyad both reported elevated symptoms in the adolescent based on predetermined cut-off scores on the SCAS and SMFQ. Follow-up actions comprised risk assessment phone calls, with adolescent participants and email notifications to parents suggesting appropriate avenues for supporting their child’s mental health.

## Results

### Randomization and Study Attrition

A total of 359 parents and 332 adolescents completed the baseline assessment. Of these, 318 parents (88.6%, 318/359) and 308 adolescents (92.8%, 308/332; this includes one adolescent who had not completed the baseline assessment) completed the postintervention assessment. This represents attrition rates of 8.9% parents (n=16) and 6.5% adolescents (n=11) from the control group and 14.0% parents (n=25) and 8.0% adolescents (n=13) from the intervention group. Figure 1 provides further details on participant flow from enrollment to postintervention assessment organized according to the CONSORT guidelines.

### Missing Data and Distributional Assumptions

Scales with a small number of items missing had these items replaced with the participant’s mean response for that scale. This led to a maximum of 23% of items (3/13) being imputed for one participant, but fewer than 11% were imputed in the remaining cases. Given the low rates of missing data at the item-level per participant, all cases with complete baseline and postintervention assessments as reflected in Figure 1 were retained for analyses. Mean imputation is acceptable when data is missing for less than 5% of cases in a dataset [43].

Model residuals for the symptom outcomes (ie, parent and child SMFQ and SCAS) at pre- and postintervention were positively skewed. Log transformation addressed this deviation from normality. For ease of interpretation, we have reported findings based on the raw data in the remaining sections because findings from analyses using the raw and transformed datasets were largely similar, except where specified below (see Multimedia Appendix 4 for MMRM analyses using transformed symptom outcome variables).

**Table 2:** Sample characteristics at baseline by intervention condition.

Participant characteristic		Intervention (N=179)	Control (N=180)	*t* or χ^2^	*P* value
**Parent sex**				0.7	.42
	Male, n (%)	26 (14.5)	20 (11.1)		
	Female, n (%)	153 (85.5)	160 (88.9)		
Parent age (years), mean (SD)		45.2 (5.26)	45.1 (5.14)	0.14	.89
**Parent marital status**				3.2	.36
	Single, n (%)	12 (6.7)	9 (5.0)		
	Married or de facto, n (%)	138 (77.1)	137 (76.1)		
	Separated or divorced, n (%)	27 (15.1)	34 (18.9)		
	Widowed, n (%)	2 (1.1)	0 (0)		
**Child sex**				0.2	.63
	Male, n (%)	102 (57.0)	97 (53.9)		
	Female, n (%)	77 (43.0)	83 (46.1)		
Child age, mean (SD)		13.7 (1.05)	13.7 (1.08)	−0.43	.67
**Family situation**				8.8	.07
	Child participant lives with both parents, n (%)	131 (73.2)	122 (67.8)		
	Parents separated but both involved in care of child participant, n (%)	21 (11.7)	36 (20.0)		
	Parents separated with only registered parent involved in care of child participant, n (%)	16 (8.9)	14 (7.8)		
	Sole parent of child participant, n (%)	10 (5.6)	4 (2.2)		
	Other	1 (0.6)	4 (2.2)		
Number of children, mean (SD)		2.37 (0.94)	2.32 (1.00)	0.45	.65
**Language**				0.01	.98
	English, n (%)	150 (83.8)	152 (84.4)		
	Other, n (%)	29 (16.2)	28 (15.6)		
**Parent employment**				1.6	.46
	Unemployed, n (%)	21 (11.7)	27 (15.0)		
	Part-time, n (%)	81 (45.3)	71 (39.4)		
	Full-time, n (%)	77 (43.0)	82 (45.6)		
**Parent studying status**				1.6	.46
	Not studying, n (%)	149 (83.2)	145 (80.6)		
	Studying part-time, n (%)	4 (2.2)	2 (1.1)		
	Studying full-time, mean (SD), n (%)	26 (14.5)	33 (18.3)		
**Parent’s highest education level, mean (SD)**				4.7	.46
	Year 7-12, n (%)	26 (14.5)	24 (13.3)		
	Trade or apprenticeship, n (%)	2 (1.1)	4 (2.2)		
	Other technical or further education (TAFE) or technical, n (%)	18 (10.1)	12 (6.7)		
	Diploma, n (%)	26 (14.5)	38 (21.1)		
	Bachelor degree, n (%)	63 (35.2)	56 (31.1)		
	Postgraduate degree, n (%)	44 (24.6)	46 (25.6)		
**Parent’s mental health diagnosis, mean (SD)**				8.3	.22
	None, n (%)	72 (40.2)	72 (40.0)		
	Past history, n (%)	60 (33.5)	77 (43.8)		
	Current diagnosis, n (%)	25 (14.0)	17 (9.4)		
	Past and current diagnosis, n (%)	20 (11.2)	13 (7.2)		
	Unanswered, n (%)	2 (1.1)	1 (0.6)		
**Child’s past mental health diagnosis, mean (SD)**				10.6	.30
	Depression, n (%)	3 (1.7)	0 (0)		
	Any anxiety disorder, n (%)	11 (6.1)	13 (7.2)		
	Autism or Asperger’s syndrome, mean (SD), n (%)	4 (2.2)	5 (2.8)		
	Other, n (%)	4 (2.2)	4 (2.2)		
	Multiple diagnoses, mean (SD), n (%)	5 (2.8)	8 (4.4)		
	No formal diagnosis, but parent concerned, n (%)	31 (17.3)	48 (26.7)		
	No past diagnosis, n (%), n (%)	105 (58.7)	88 (48.9)		
	Unanswered, n (%)	16 (8.9)	14 (7.8)		
**Child’s current mental health diagnosis**				5.4	.50
	Depression, n (%)	0	1 (0.6)		
	Any anxiety disorder, n (%)	13 (7.3)	13.0 (7.2)		
	Autism or Asperger’s^a^ syndrome, n (%)	3 (1.7)	4 (2.2)		
	Other, n (%)	6 (3.4)	3 (1.7)		
	Multiple diagnoses, n (%)	12 (6.7)	13 (7.2)		
	No formal diagnosis, but parent concerned, n (%)	38 (21.2)	52 (28.9)		
	No diagnosis, n (%)	104 (58.1)	90 (50.0)		
	Unanswered, n (%)	3 (1.7)	4 (2.2)		

^a^Two children who were reported by their parents to have a past diagnosis of autism or Asperger’s syndrome were categorized under “Multiple diagnosis” as they also had another current mental health diagnosis.

### Baseline Sample Characteristics, Attrition, and Symptom Elevation Follow-Up

Parent and child participants did not differ significantly between the two conditions on any of the outcome measures or demographic variables assessed at baseline. As shown in Table 2, most parent participants were female, married, or in a de facto relationship; living with the adolescent participant; and employed at least part-time. Almost 60% had attained graduate or postgraduate qualifications and reported either a past and/or current mental health diagnosis. Just over half of the adolescents were male, and most adolescents were reported by their parents to have no prior or current mental health diagnosis.

Adherence to the intervention and attrition rates for the follow-up assessment did not differ significantly between conditions and were not related to any participant characteristics at baseline except for parent age. Parents within dyads with missing postintervention data were younger than those with available postintervention data.

We also examined whether follow-up actions taken in response to elevations in adolescent depression and anxiety symptoms differed between conditions, as these may have impacted on reported outcomes at postintervention. Chi-square tests indicated no significant differences between conditions in the proportion of follow-up actions undertaken (*p* s>.05).

### Time Interval Between Baseline and Postintervention Assessment Completion

The mean time interval between parent baseline and postintervention assessment completions was 118 days (SD=34.4, median=105, range=86-279). The mean time interval between child baseline and postintervention assessment completions was 110 days (SD=28.0, median=99, range=85-277 days). These time intervals did not differ significantly between conditions. The wide interval ranges were due to programming errors whereby automated postintervention assessment alerts for the research team to follow up with 59 dyads were not delivered. The error was detected during an audit of participant numbers on August 1, 2016, which also indicated that the follow-up assessment date for another dyad had been missed because of human error. Of the 60 dyads, 59 were contacted to complete the postintervention assessment; the remaining one was not contacted for this assessment as their final (12-month) follow-up assessment date was too near to this time (ie, within a month). The proportion of participants who were affected by this technical error did not differ between conditions.

### Time Interval Between Parent and Child Assessment Completions at Each Assessment

For baseline assessments, the mean time interval between child and parent assessment completions, for dyads where both were completed (n=332) was 4.88 days (SD=8.62, range=0-76). For postintervention assessments, time intervals between completed child and parent assessments (n=294) averaged 11.4 days (SD=21.2, range=0-167). The extreme larger ends of these ranges were because of a small number of parent participants being difficult to contact during extended school holiday periods.

### Intervention Completion and Adherence Rates Within the Whole Sample

At the time of data extraction, the average intended program usage within the intervention group (n=179) was 6.85 out of the nine modules available for selection in the personalized program. The average observed usage within the intervention group was 5.17 modules. Participants in the intervention group completed an average of 73.7% of their locked-in program. Intervention adherence within the intervention group was 44.1% (n=79). In addition, 15.1% of the intervention group (n=27) completed modules not initially selected as part of their personalized programs (average of 2 modules). Intervention adherence within the control group (n=180) was 72.8% (n=131).

### Intervention Completion and Adherence Rates Within Follow-Up Sample

Of the 318 parents who completed the postintervention assessment, 165 (control=108, intervention=57) completed 100% of their program during the active intervention phase, which was defined as the time between completion of parent baseline assessment and completion of child postintervention assessment (a parent’s follow-up completion timestamp was used if their child did not complete the postintervention assessment). Of the 308 child participants who completed the postintervention assessment, the parents of 159 (control=106, intervention=53) had completed their programs before completing their follow-up assessments.

A further 14 parent participants from the intervention group who completed their postintervention assessment did not have completion timestamps on some of their completed modules in their program, most likely because of technical error. Consequently, we were unable to conclusively determine whether they had completed their programs within the active intervention phase. There were 8 adolescent participants from this group who completed the postintervention assessment.

There were 23 parents (control=17, intervention=6) who completed their program after the active intervention phase. At the time of their follow-up assessment, intervention group parents in this subsample had completed an average of 53.0% of their program, whereas the average completion rate was 70.6% for control group parents.

The remaining 116 parent participants (control=39, intervention=77) who completed the postintervention assessment still had not completed their whole program at the time their data was extracted for analyses. In this subgroup, the average intervention completion rates were 61.9% and 68.7%, respectively, for intervention and control group parents.

### Primary Outcome Analysis

#### Parenting to Reduce Adolescent Depression and Anxiety Scale (PRADAS), Parent-Report

**Figure 2:**
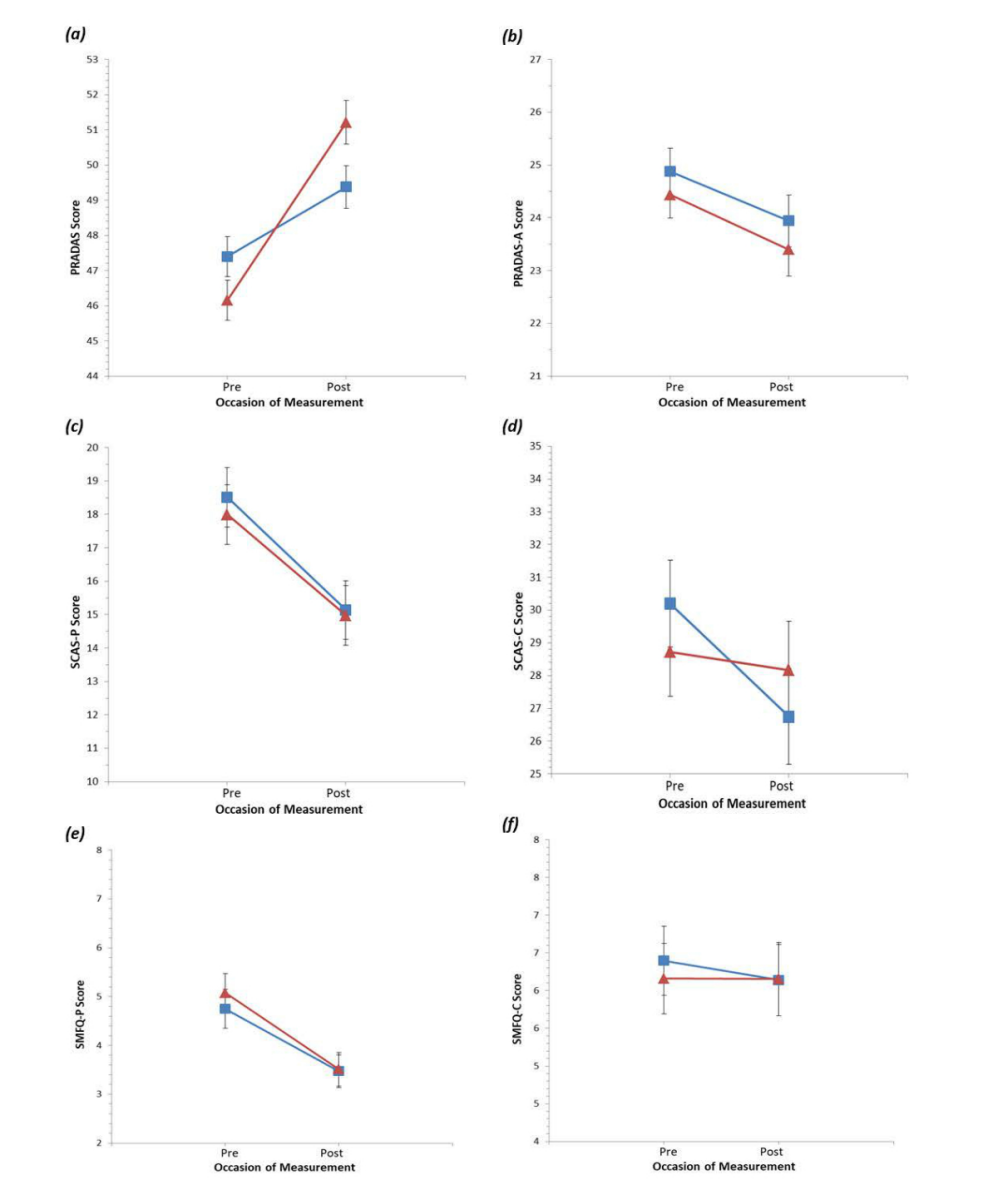
Graphs of estimated marginal means of primary and secondary outcomes for each intervention condition and occasion of measurement. Error bars represent standard error. Square=control, triangle=intervention.

We found a significant interaction between condition and time on total PRADAS scores, Cohen *d*=0.57, 95% CI 0.34-0.79. The intervention group’s mean PRADAS score significantly exceeded that of the control group at postintervention, producing a small effect (see Table 3 and Figure 2; observed mean and SD are presented in Multimedia Appendix 4).

### Secondary Outcome Analyses

#### Adolescent Anxiety and Depression

There were no significant interactions between condition and time on the SMFQ-P, SMFQ-C, and SCAS-P scores. Across conditions, parent participants reported significantly decreased symptoms of depression and anxiety from baseline to post intervention (Table 3).

A condition by time interaction on adolescent-reported anxiety was observed (Table 3; see also Figure 2), and individual comparisons revealed that self-reported anxiety decreased at postintervention only for the control group, with no change in the intervention group (Table 3). However, this interaction was no longer significant when the transformed SCAS-C data was analyzed; instead, both conditions showed significant decreases in symptoms of anxiety from baseline to postintervention (see Multimedia Appendix 4).

#### Parental Concordance With the Parenting Guidelines, Adolescent-Report

There was no significant condition by time interaction on the PRADAS-A total score. Instead, both groups reported significantly reduced parental concordance with the parenting guidelines from baseline to post intervention.

### Post-Hoc Moderation Analyses

#### Parental Concordance With the Parenting Guidelines, Parent-Report

Post-hoc analyses revealed that adolescent-report depression and anxiety symptoms at baseline did not significantly moderate intervention effects on change in PRADAS scores from baseline to post intervention, *F*_1,294_=3.59, *P*=.059, accounting for 1% variance. Conditional effects analyses revealed that among parents whose adolescents reported lower (SCAS-C≤12.42) and average (12.42<SCAS-C<46.68) levels of anxiety at baseline, PiP led to greater increases in PRADAS scores compared with the control condition (see Multimedia Appendix 4). The difference between conditions was not significant for parents whose adolescents reported higher levels of baseline anxiety (SCAS-C≤46.68).

#### Parental Concordance With the Parenting Guidelines, Adolescent-Report

Moderation analyses revealed that parent-reported adolescent anxiety moderated intervention effects on adolescent-report parenting, *F*_1,303_=20.09, *P*<.001, accounting for 6.2% variance in change in PRADAS-A scores. Among parents who reported lower levels of adolescent anxiety at baseline (SCAS-P≤6.04), adolescents whose parents received PiP reported no change in PRADAS-A scores, whereas adolescents whose parents received the control intervention reported a reduction in PRADAS-A scores, *d*=0.50 (95% CI 0.27-0.72). However, among parents who reported higher levels of adolescent anxiety at baseline (SCAS-P≥29.30), the opposite pattern was observed, with PRADAS-A scores remaining stable in the control condition but decreasing in the PiP condition, *d*=−0.53 (95% CI −0.76 to −0.31]. No significant difference between conditions was observed at mean levels of parent-reported adolescent anxiety. Parent-reported adolescent depressive symptom levels at baseline did not moderate intervention effects on PRADAS-A scores. See Multimedia Appendix 4 for specific conditional effects referred to above.

**Table 3:** Estimates of marginal means (EMM), standard errors (SE), and mixed model repeated measures analyses of primary and secondary outcome scores at baseline and postintervention time points. There were no significant differences between conditions on any of the primary or secondary outcome measures at baseline.

Outcome measure and time		Outcome scores		*F*^a^	df	*P* value	*d*_post_^b^ (95% CI)	*d*_interaction_^c^ (95% CI)
		Intervention EMM (SE)	Control EMM (SE)					
**PRADAS^d^**								
	Baseline	46.40 (0.57)	47.40 (0.57)	25.54	1, 320	<.001	0.27 (0.05-0.49)	0.57 (0.34-0.79)
	Postintervention	51.21 (0.62)	49.38 (0.61)					
**PRADAS-A^e^**								
	Baseline	24.44 (0.44)	24.88 (0.43)	0.04	1, 308	.835	−0.11 (−0.34 to 0.11)	0.02 (−0.20 to 0.25)
	Postintervention	23.40 (0.50)	23.94 (0.49)					
**SCAS-P^f^**								
	Baseline	17.99 (0.90)	18.51 (0.89)	0.16	1, 327	.693	0.04 (−0.18 to 0.26)	0.04 (−0.18 to 0.26)
	Postintervention	14.98 (0.89)	15.14 (0.88)					
**SCAS-C^g^**								
	Baseline	28.73 (1.36)	30.20 (1.33)	5.08	1, 306	.025^h^	0.09 (−0.14 to 0.31)	0.26 (0.03-0.48)
	Postintervention	28.17 (1.50)	26.75 (1.43)					
**SMFQ-P^i^**								
	Baseline	5.07 (0.40)	4.75 (0.40)	0.33	1, 333	.566	0.05 (−0.17 to 0.27)	0.06 (−0.16 to 0.28)
	Postintervention	3.51 (0.34)	3.47 (0.34)					
**SMFQ-C^j^**								
	Baseline	6.16 (0.47)	6.40 (0.46)	0.26	1, 308	.609	0.04 (−0.19 to 0.26)	0.06 (−0.17 to 0.28)
	Postintervention	6.16 (0.49)	6.14 (0.48)					

^a^Test of the condition by time interaction.

^b^Cohen *d* effect size of difference between conditions at postintervention, reported with 95% CI in parentheses.

^c^Cohen *d* effect size of interaction, reported with 95% CI in parentheses.

^d^PRADAS: Parenting to Reduce Adolescent Depression and Anxiety Scale.

^e^PRADAS-A: Parenting to Reduce Adolescent Depression and Anxiety Scale- Adolescent.

^f^SCAS-P: *Spence Children’s Anxiety Scale*-Parent version.

^g^SCAS-C: *Spence Children’s Anxiety Scale*-Child version.

^h^This effect became nonsignificant when the analysis was run using log-transformed data to correct the skewed distribution.

^i^SMFQ-P: Short Moods and Feelings Questionnaire-Parent version.

^j^SMFQ-C: Short Moods and Feelings Questionnaire-Child version.

#### Adolescent Anxiety and Depression

Adolescent-report baseline depressive symptoms had a significant moderation effect on change in SMFQ-P scores,  *F*_1,294_=5.97, *P*=.015, accounting for 1.9% variance. Conditional effects analyses revealed that change in SMFQ-P scores were significantly different between conditions only among adolescents who had reported higher levels of depressive symptoms at baseline (SMFQ-C≥12.19), with parents who received PiP reporting greater symptom reduction than those who received the control intervention, *d*=−0.35 (95% CI −0.58 to −0.12; see Multimedia Appendix 4). No significant moderation effects were found for changes in SMFQ-C, SCAS-C, and SCAS-P.

## Discussion

### Principal Findings: Primary Outcome

This RCT evaluated the short-term effects of the PiP intervention, a Web-based parenting intervention to prevent adolescent depression and anxiety. As hypothesized, compared with an educational-factsheet control intervention, PiP was found to produce greater improvements from baseline to post intervention (3 months later) in self-reported parenting behaviors, indicating increased concordance with evidence-based Parenting Guidelines [28]. This represented a medium effect size, although the intervention and control group difference at postintervention was a small effect. The parenting behaviors assessed represent parental risk and protective factors for adolescent depression and anxiety [12,29,34]; hence the current findings suggest that by modifying these parenting behaviors, PiP may have the potential to confer protection against adolescent depression and anxiety over the long term.

### Principal Findings: Secondary Outcomes

Three months after commencing the intervention, no corresponding reductions in adolescent depressive and anxiety symptoms were found in secondary outcome analyses based on either parent or adolescent report. There was also no significant improvement in parenting based on adolescent report; in fact, across the sample, adolescents reported a slight reduction in guidelines-concordant parenting behaviors over the 3 months between the baseline and postintervention assessments. The lack of demonstrable effects of PiP on adolescent-reported parenting suggests that the parent-reported improvements in parenting were not noticed or not interpreted as such by their adolescents. This may reflect difficulties inherent in assessing early adolescents’ perceptions of parenting, as they may rely on more generalized impressions of their parent’s parenting and may not have the capacity to distinguish between specific, recent behaviors from general parenting practices over time [44]. Other possibilities are that adolescents may need more time before they notice improvements in their parents’ parenting, or that parents’ perceived changes in parenting did not translate into tangible behavioral changes. Parent-adolescent divergence in perceptions of parenting, especially in early adolescence, is normative and well-established in the developmental literature [45]. Developmental perspectives posit that adolescents’ maturation processes, including autonomy seeking and individuation, give rise to a period of time when parents and adolescents experience the same interactions differently, which in turn account for the divergence in parent-adolescent perceptions of parenting [46]. Long-term follow-up and further research is required to elucidate the associations between these short-term perceptions of parenting and long-term adolescent depression and anxiety outcomes.

### Post-Hoc Moderation Effects

As a universal prevention program involving a primarily community-based sample, we found that PiP did not demonstrate any short-term effect on adolescent depressive or anxiety symptoms, likely due to a “floor effect” (symptom levels were low at baseline for most participants). Interestingly, post-hoc moderation analyses suggest that PiP did have a significant effect on reducing parent-reported adolescent depressive symptoms for adolescents who had reported higher levels of symptoms at baseline. This suggests that PiP may be useful as an indicated intervention for parents of adolescents with elevated depressive symptoms. Compared with an active-control condition, PiP produced greater improvements in parent-reported parenting regardless of adolescent baseline depressive or anxiety symptoms. Post-hoc findings involving changes in adolescent-reported parenting are more challenging to interpret, suggesting that the extent to which adolescents perceived a reduction in guidelines-concordant parenting behaviors depended on the adolescents’ parent-reported anxiety symptoms at baseline. Among adolescents with lower anxiety levels at baseline, those whose parents received PiP (as opposed to the control intervention) perceived less reduction in guidelines-concordant parenting. On the other hand, among adolescents with higher anxiety levels at baseline, those whose parents received PiP perceived greater reduction in guideline-concordant parenting. Generally, although these post-hoc findings are promising because of the exploratory nature of these analyses and the inconsistencies across measures, future research is required to test the efficacy of PiP as an indicated prevention intervention in a rigorously designed RCT.

### Intervention Engagement

PiP was generally well received by parents, with a reasonably high mean proportion of intervention completion (74% of their personalized program, which comprised an average of 6.85 modules), with 15% of parents completing additional modules outside of their initially selected program. The current RCT did not include a comparison group which received the online PiP without phone support; hence, we cannot determine whether the phone calls aided in the intervention completion rates. However, the rates from a five-arm RCT of Web-based preventive interventions for adults [47] provide a basis for inferring that the phone calls contributed to higher rates of intervention completion. Specifically, that trial included three intervention arms (active website, active website with email, and active website with telephone) that achieved intervention completion rates of 37%, 55%, and 73%, respectively. Coincidentally, the proportion of PiP completion, which was supported by weekly administrative reminder phone calls, was very similar to the “active website with telephone” arm of the earlier RCT. If PiP was implemented without phone support, which conveys a sense of accountability [48], it is possible that completion rates may be closer to 40%. Nonetheless, qualitative feedback from parents, provided spontaneously through the weekly phone calls, indicate that most parents found PiP to be engaging and that the strategies provided were practical and useful. Some parents found it overwhelming to receive their personalized feedback report after completing their baseline assessment (about 45-min long) and then be required to start their first module immediately thereafter. In a real-world setting, this may not be such an issue as only the PRADAS, which takes less than 15 min to complete [34], would be required to generate the tailoring of PiP. Some parents, especially where there are existing challenges with the family situation (eg, heightened family conflict) or adolescent mental health, also sought more support and information through the phone calls. This suggests that if implemented as an indicated prevention program, phone support may need to be tailored to the parents’ level of need.

### Strengths and Limitations

This study had several notable strengths. It recruited a large community-based sample of parents and early adolescents and achieved a relatively low attrition rate for an online intervention at 3-month follow-up. Parents’ engagement in their allocated intervention was acceptable in the intervention group and high in the control group. One methodological strength was the utilization of an active-control comparison group, which allowed us to test whether the PiP intervention yields benefits beyond simply involving parents in a parenting intervention [49]. Another strength was the inclusion of both parent and adolescent informants of parenting and adolescent symptoms, which permitted an exploration of outcome-reporting bias by parents who were aware of their group allocation; it also enabled analytic models (eg, in post-hoc analyses) that attempted to minimize shared method variance.

Nonetheless, there are limitations to this RCT. First, it recruited a large proportion of self-selected parents who were mostly mothers, highly educated, from intact families, and did not speak a language other than English at home. Although such sociodemographic characteristics are typical of adult participants in other online interventions [50] and are similar to a preventive online parenting intervention for preschool children [51], further research is required to determine whether PiP would have similar effects for families with different characteristics. Second, for reasons of parsimony, the current RCT only included one parent and one adolescent per family. In future research and outside the research context, it is possible for both parents to receive their own personalized intervention, which can help both of them become more concordant with the recommendations of the Parenting Guidelines and coparent more effectively. Existing evidence from programs that examined the unique effects of father and mother engagement in parenting interventions [52] suggests that including both parents could in turn enhance the effects of the intervention on child outcomes. Third, to reduce participant burden, we relied on new parenting measures developed specifically for this study, to the exclusion of other validated parenting measures, especially by adolescents who are subject to less outcome-reporting bias as they are less likely to be aware of their parent’s intervention assignment. One implication of this is, for example, that we could not test whether the nonsignificant intervention effect on adolescent-report parenting is because of the insensitivity of the new PRADAS-A measure to detect changes in parenting, or broader developmental factors as discussed above [44-46]. We also did not collect observational data on parent-adolescent interactions, which would be useful for verifying self-reported changes in parenting. Although we used an active-control intervention, we did not assess or control for differential motivations and expectations in parents receiving each intervention (it is clear that most parents in the control condition wanted to receive PiP instead), hence, limiting our ability to draw firm causal conclusions [49]. Moreover, the control condition was not ideally matched in terms of interactivity and the amount of administrative support received (ie, on average, parents in this condition received fewer weekly phone calls than those in the intervention group). It was also not possible to determine whether parents in the control condition actually completed their intervention (ie, read all their factsheets). The “completion” of a factsheet may be better matched to the “completion” of a module if, for example, parents in the control condition were required to click on a button to “Finish session,” just as parents in the intervention condition were required to click on a “Finish module” button. Future evaluations of PiP should also include other measures of adolescent functioning outcomes (eg, quality of life, emotion regulation, and school engagement) and more broadly, measures of cost-effectiveness.

### Comparison With Prior Work

To the best of our knowledge, PiP is the first tailored Web-based parenting intervention for the prevention of adolescent depression and anxiety. A recent systematic review of RCTs of parenting interventions to prevent internalizing problems in children in the age range of 0 to 18 years revealed a dearth of rigorously evaluated, evidence-based interventions for parents of adolescents [9]. Of the 51 programs included in the review, only three were designed for parents of adolescents [53-55]. Moreover, despite the potential value of Web-based delivery for universal prevention programs [22,24], none of these three existing interventions are Web-based, and only one of these, the Tuning in to Teens (TINT; [54]) program, was found to successfully improve parenting and reduce adolescent anxiety (based on both parent and adolescent report) and depressive symptoms (based on parent report only) at approximately 9 months post intervention. Specifically, compared with a no-intervention control condition, TINT yielded a moderately large effect size on the change over time in parent-report parenting (Cohen *d*=0.76). Considering the differences in the control condition (no-intervention vs active-control) and modality (face-to-face vs Web-based), PiP’s effects on parenting compare favorably with TINT’s.

A limitation of existing preventive parenting programs is their focus on one or only a few parenting risk and protective factors for adolescent depression and anxiety [9]. As parents differ in their areas of competence and difficulties, such a narrow-focus approach may mean that these programs do not adequately address the range of modifiable risk and protective parenting factors for adolescent depression and anxiety that are relevant for each parent or family. As an automatically tailored program, PiP addresses this limitation by screening each parent across all evidence-based parenting risk and protective factors to provide a more thorough coverage of areas that may be important to target in preventive intervention for the particular parent.

### Conclusions

The current RCT found that compared with an active-control condition, PiP produced greater short-term improvements in parental risk and protective factors for adolescent depression and anxiety from the parents’ perspective, which represents the most proximal outcome. Moreover, the intervention was well received by parents. It remains to be ascertained, in longer-term follow-up (a 12-month follow-up assessment has commenced), whether the self-reported improvements in parenting will translate into corresponding adolescent-reported improvements and in turn give rise to protection against depression and anxiety in the adolescent. Given the brevity of the intervention and its fully developed Web-delivery modality, these preliminary findings are promising and suggest that PiP may be useful as a low-cost, scalable, and sustainable public health universal prevention program to empower parents in their parenting of adolescents.

## Appendix

Multimedia Appendix 1 CONSORT‐EHEALTH checklist (V 1.6.1).

Multimedia Appendix 2 Participants’ informed consent documentation.

Multimedia Appendix 3 Screenshots of the Partners in Parenting intervention.

Multimedia Appendix 4 Observed means and standard deviations of outcome variables, results of mixed effect model repeat measurements (MMRMs) using transformed data, and posthoc analyses.

## Abbreviations

EMM: estimates of marginal means
MMRM: mixed model of repeated measures
PiP: Partners in Parenting
PRADAS: Parenting to Reduce Adolescent Depression and Anxiety Scale
PRADAS-A: Parenting to Reduce Adolescent Depression and Anxiety Scale-Adolescent version
PSD: Persuasive Systems Design
RCT: randomized controlled trial
SCAS: Spence Children’s Anxiety Scale
SCAS-C: Spence Children’s Anxiety Scale-Child version
SCAS-P: Spence Children’s Anxiety Scale-Parent version
SMFQ: Short Mood and Feelings Questionnaire
SMFQ-C: Short Mood and Feelings Questionnaire-Child version
SMFQ-P: Short Mood and Feelings Questionnaire-Parent version
TINT: Tuning in to Teens

## References

[1] Kessler RC, Petukhova M, Sampson NA, Zaslavsky AM, Wittchen H (2012). Twelve-month and lifetime prevalence and lifetime morbid risk of anxiety and mood disorders in the United States. Int J Methods Psychiatr Res.

[2] Lawrence D, Johnson S, Hafekost J, Boterhoven DHK, Sawyer M, Ainley J (2015). Department of Health.

[3] Rao U, Ryan ND, Birmaher B, Dahl RE, Williamson DE, Kaufman J, Rao R, Nelson B (1995). Unipolar depression in adolescents: clinical outcome in adulthood. J Am Acad Child Adolesc Psychiatry.

[4] Last CG, Hansen C, Franco N (1997). Anxious children in adulthood: a prospective study of adjustment. J Am Acad Child Adolesc Psychiatry.

[5] Andrews G, Issakidis C, Sanderson K, Corry J, Lapsley H (2004). Utilising survey data to inform public policy: comparison of the cost-effectiveness of treatment of ten mental disorders. Br J Psychiatry.

[6] Patel V, Flisher A, Hetrick S, McGorry P (2007). Mental health of young people: a global public-health challenge. Lancet.

[7] Fisak BJ, Richard D, Mann A (2011). The prevention of child and adolescent anxiety: a meta-analytic review. Prev Sci.

[8] Merry SN, Hetrick SE, Cox GR, Brudevold-Iversen T, Bir JJ, McDowell H (2011). Psychological and educational interventions for preventing depression in children and adolescents. Cochrane Database Syst Rev.

[9] Yap MB, Morgan AJ, Cairns K, Jorm AF, Hetrick SE, Merry S (2016). Parents in prevention: a meta-analysis of randomized controlled trials of parenting interventions to prevent internalizing problems in children from birth to age 18. Clin Psychol Rev.

[10] Beesdo K, Knappe S, Pine DS (2009). Anxiety and anxiety disorders in children and adolescents: developmental issues and implications for DSM-V. Psychiatr Clin North Am.

[11] Cairns KE, Yap MB, Pilkington PD, Jorm AF (2014). Risk and protective factors for depression that adolescents can modify: a systematic review and meta-analysis of longitudinal studies. J Affect Disord.

[12] Sandler IN, Schoenfelder EN, Wolchik SA, MacKinnon DP (2011). Long-term impact of prevention programs to promote effective parenting: lasting effects but uncertain processes. Annu Rev Psychol.

[13] Yap MB, Pilkington PD, Ryan SM, Jorm AF (2014). Parental factors associated with depression and anxiety in young people: a systematic review and meta-analysis. J Affect Disord.

[14] Yap MB, Jorm AF (2011). Parents' beliefs about actions they can take to prevent depressive disorders in young people: results from an Australian national survey. Epidemiol Psychiatr Sci.

[15] Fisak BJ, Richard D, Mann A (2011). The prevention of child and adolescent anxiety: a meta-analytic review. Prev Sci.

[16] Hetrick SE, Cox GR, Witt KG, Bir JJ, Merry SN (2016). Cognitive behavioural therapy (CBT), third-wave CBT and interpersonal therapy (IPT) based interventions for preventing depression in children and adolescents. Cochrane Database Syst Rev.

[17] Heinrichs N, Bertram H, Kuschel A, Hahlweg K (2005). Parent recruitment and retention in a universal prevention program for child behavior and emotional problems: barriers to research and program participation. Prev Sci.

[18] Mrazek P, Haggerty R (1994). Reducing Risks for Mental Disorders: Frontiers for Preventive Intervention Research.

[19] Rose G (1992). The Strategy of Preventive Medicine.

[20] Koerting J, Smith E, Knowles MM, Latter S, Elsey H, McCann DC, Thompson M, Sonuga-Barke EJ (2013). Barriers to, and facilitators of, parenting programmes for childhood behaviour problems: a qualitative synthesis of studies of parents' and professionals' perceptions. Eur Child Adolesc Psychiatry.

[21] (2014). Australian Bureau of Statistics.

[22] Cuijpers P, van Straten A, Warmerdam L, van Rooy M (2010). Recruiting participants for interventions to prevent the onset of depressive disorders: possible ways to increase participation rates. BMC Health Serv Res.

[23] Metzler CW, Sanders MR, Rusby JC, Crowley RN (2012). Using consumer preference information to increase the reach and impact of media-based parenting interventions in a public health approach to parenting support. Behav Ther.

[24] Yap MB, Martin PD, Jorm AF (2017). Online parenting guidelines to prevent adolescent depression and anxiety: evaluating user characteristics and usefulness. Early Interv Psychiatry.

[25] Andrews G, Cuijpers P, Craske MG, McEvoy P, Titov N (2010). Computer therapy for the anxiety and depressive disorders is effective, acceptable and practical health care: a meta-analysis. PLoS One.

[26] Love SM, Sanders MR, Turner KM, Maurange M, Knott T, Prinz R, Metzler C, Ainsworth AT (2016). Social media and gamification: engaging vulnerable parents in an online evidence-based parenting program. Child Abuse Negl.

[27] Yap MB, Lawrence KA, Rapee RM, Cardamone-Breen MC, Green JM, Jorm AF (2017). Partners in parenting: a multi-level web-based approach to support parents in prevention and early intervention for adolescent depression and anxiety. JMIR Ment Health.

[28] (2013). Parenting Strategies Program.

[29] Jorm AF (2015). Using the Delphi expert consensus method in mental health research. Aust N Z J Psychiatry.

[30] Sanders MR, Kirby JN (2012). Consumer engagement and the development, evaluation, and dissemination of evidence-based parenting programs. Behav Ther.

[31] Oinas-Kukkonen H, Harjumaa M (2009). Persuasive systems design: key issues, process model, and system features. CAIS.

[32] Kelders SM, Kok RN, Ossebaard HC, Van Gemert-Pijnen JE (2012). Persuasive system design does matter: a systematic review of adherence to web-based interventions. J Med Internet Res.

[33] Kreuter M, Farrell D, Olevitch L, Brennan L (2000). Tailoring Health Messages: Customizing Communication With Computer Technology.

[34] Cardamone-Breen MC, Jorm AF, Lawrence KA, Mackinnon AJ, Yap MB (2017). The parenting to reduce adolescent depression and anxiety scale: assessing parental concordance with parenting guidelines for the prevention of adolescent depression and anxiety disorders. PeerJ.

[35] (2010). Parenting Strategies Program.

[36] Raising Children Network.

[37] Angold A, Costello E, Messer S (1995). Development of a short questionnaire for use in epidemiological studies of depression in children and adolescents. Int J Methods Psychiatr Res.

[38] Spence SH (1997). Structure of anxiety symptoms among children: a confirmatory factor-analytic study. J Abnorm Psychol.

[39] Nauta MH, Scholing A, Rapee RM, Abbott M, Spence SH, Waters A (2004). A parent-report measure of children’s anxiety: psychometric properties and comparison with child-report in a clinic and normal sample. Behav Res Ther.

[40] Kelders SM, Kok RN, Ossebaard HC, Van Gemert-Pijnen JE (2012). Persuasive system design does matter: a systematic review of adherence to web-based interventions. J Med Internet Res.

[41] Gueorguieva R, Krystal JH (2004). Move over ANOVA: progress in analyzing repeated-measures data and its reflection in papers published in the Archives of General Psychiatry. Arch Gen Psychiatry.

[42] Hayes AF (2012). AFHayes.com.

[43] Bell ML, Fairclough DL (2014). Practical and statistical issues in missing data for longitudinal patient-reported outcomes. Stat Methods Med Res.

[44] Branje SJ, van Aken MA, van Lieshout CF (2002). Relational support in families with adolescents. J Fam Psychol.

[45] Branje S, Laursen B, Collins W (2012). Parent-child communication during adolescence. Routledge handbook of family communication. 2nd edition.

[46] Collins W, Laursen B (2000). Adolescent relationships: The art of fugue. Close Relationships: A Sourcebook.

[47] Christensen H, Batterham P, Mackinnon A, Griffiths KM, Kalia HK, Kenardy J, Gosling J, Bennett K (2014). Prevention of generalized anxiety disorder using a web intervention, iChill: randomized controlled trial. J Med Internet Res.

[48] Mohr DC, Cuijpers P, Lehman K (2011). Supportive accountability: a model for providing human support to enhance adherence to eHealth interventions. J Med Internet Res.

[49] Boot WR, Simons DJ, Stothart C, Stutts C (2013). The pervasive problem with placebos in psychology: why active control groups are not sufficient to rule out placebo effects. Perspect Psychol Sci.

[50] Murray E (2012). Web-based interventions for behavior change and self-management: potential, pitfalls, and progress. Med 2 0.

[51] Morgan AJ, Rapee RM, Salim A, Goharpey N, Tamir E, McLellan LF, Bayer JK (2017). Internet-delivered parenting program for prevention and early intervention of anxiety problems in young children: randomized controlled trial. J Am Acad Child Adolesc Psychiatry.

[52] Frank TJ, Keown LJ, Sanders MR (2015). Enhancing father engagement and interparental teamwork in an evidence-based parenting intervention: a randomized-controlled trial of outcomes and processes. Behav Ther.

[53] Bearslee WR, Wright EJ, Gladstone TR, Forbes P (2007). Long-term effects from a randomized trial of two public health preventive interventions for parental depression. J Fam Psychol.

[54] Kehoe CE, Havighurst SS, Harley AE (2013). Tuning in to teens: improving parent emotion socialization to reduce youth internalizing difficulties. Soc Dev.

[55] Rotheram-Borus MJ, Stein JA, Lester P (2006). Adolescent adjustment over six years in HIV-affected families. J Adolesc Health.

