# Supplementary material for: A Tailored Web-Based Intervention to Improve Parenting Risk and Protective Factors for Adolescent Depression and Anxiety Problems: Postintervention Findings From a Randomized Controlled Trial
Source: J Med Internet Res. 2018 Jan 19;20(1):e17. doi: 10.2196/jmir.9139 (PMC5797292; doi:10.2196/jmir.9139)
Supplement: Multimedia Appendix 2 [file jmir_v20i1e17_app2.pdf]

Appendix 2: Participant Informed Consent Documentation

MONASH University

PARENT EXPLANATORY STATEMENT

Partners in Parenting: Evaluating an online personalised parenting program

Chief Investigator: Dr Marie Yap, School of Psychological Sciences, Monash University. Phone: (03) 9905 0723, email: marie.yap@monash.edu
Student Researchers: Jacqueline Green, Doctor of Philosophy candidate; Claire Nicolas, Doctor of Psychology (Clinical) candidate; Peter Martin, Honours student; Brooke Swierzbiolek, Honours student; School of Psychological Sciences, Faculty of Medicine, Nursing and Health Sciences Monash University. Phone: (03) 9905 1250, email: med-partnersinparenting@monash.edu
Senior Research Officer: Shireen Mahtani, School of Psychological Sciences, Monash University. Phone: (03) 9905 1250, email: med-partnersinparenting@monash.edu

Invitation to participate in research
You are invited to take part in research being conducted by Monash University as part of a Research by Higher Degree (PhD), a Doctorate of Psychology (Clinical) and Psychology Honours theses. Please read this Explanatory Statement in full before deciding whether or not to participate. If you would like further information regarding any aspect of the project, please contact the researchers via the phone numbers or email addresses listed above.

What does the research involve?
This study aims to evaluate whether an online program providing parents with personalised information and tips on parenting strategies is effective in improving parents' knowledge about teenage depression and anxiety and parenting behaviours, hence preventing or reducing the impact of depression and anxiety in their teenager.

What will I be asked to do?
If you agree to participate, you will be asked to do the following:

1. Go online to www.partnersinparenting.net.au
   Fill in the online registration and consent form, and indicate that you have discussed the project with your teenager and that they agree to take part too. You will then need to click on a button to indicate that you have discussed the project with your teenager and they agree to take part too. You will be asked to provide brief demographic information so that we can ensure you are eligible for this study. We will also ask you to provide a telephone number that we can contact your teenager on at a preferred time and day of the week. If you consent for both you and your teenager to participate, click on 'both my teenager and I agree to participate in this study'. You will be emailed a copy of the youth explanatory statement to give to your teenager.

2. Your teenager will be contacted by phone by a member of the research team from the Monash University School of Psychological Sciences. The purpose of the phone call is to discuss with your teenager the explanatory statement to ensure they understand and agree to take part in the research. Your teenager can decide whether or not to take part in the study when the researcher contacts them. If your teenager agrees to participate, the researcher will give them their own login details during the phone call, and provide any guidance to them as needed, to complete their online assessment.
   The online assessment includes questions about your teenager's experience of your current parenting practices, and different feelings and behaviours that may be associated with depression and anxiety (about 30 minutes altogether). Your teenager will be asked to complete the same online assessment again 3 and 12 months later. We ask you to encourage your teenager to complete these because their perspective is very important.

If you and/or your teenager report high levels of difficulty with your teenager's mood or anxiety, a member of the research team will contact you and your teenager to ensure that your teenager gets the support that they need.

3. You will then receive an email requesting that you complete an online assessment asking about your current parenting practices regarding your teenager, as well as questions about feelings and behaviours in your teenager that may be associated with depression and anxiety (50-65 minutes altogether).
4. You will then be randomly allocated to one of two groups where you will receive either:
   a) The *Guidelines,* a personalised feedback report, and the online parenting program comprising up to 9 modules which are recommended for you based on your survey responses. Each module may take between 15-25 minutes to complete, depending on the topic and the way you choose to engage with it.

   OR

   b) Five weekly emails linking you to your online factsheet for the week.

   Allocation to each group is automated by a computer program. You will find out which group you have been allocated to once you have completed your first online assessment.
5. You will receive a phone call from a research staff member once a week until you have completed your allocated program. Each call will last approximately 5-10 minutes. These calls provide you with an opportunity to ask any questions related to the study procedures (e.g. trouble logging into the website), but will not provide any therapeutic support.
6. You and your teenager (if they agree to take part) will be contacted again 3 months and 1 year later, to complete similar follow-up online surveys (30 minutes each). These surveys will be delivered in the same format as the initial survey.

All parts of this study can be completed at any time or place of convenience to you, as long as there is internet access.

Who is being asked to participate?
We are inviting families with at least one teenager aged between 12 and 15 years (inclusive) who live in Australia, have regular access to the internet, and are proficient in English, to participate in this research. One parent and one teenager (aged 12-15 years) per family are invited to participate.

Source of funding
This research project has been funded by Australian Rotary Health.

What if I change my mind?
Participation in this research is completely voluntary. If you decide to participate, you will be asked to check the relevant boxes in the online consent form. If you change your mind at any stage, you are free to withdraw from the research (e.g. you may cease to complete the modules or surveys). You may also request that your data (i.e. survey responses) be withdrawn once submitted, prior to the final report being written. After this time, you will not be able to withdraw your data.

What are the benefits?
This project is designed to help equip parents with parenting strategies that can reduce depression and anxiety in their teenagers. In the longer term, it is hoped that the program will reduce teenage depression and anxiety problems in the community.

Are there any risks?
The risks of participating in this research are low; however we have identified the following potential risks:

1. Although unlikely, it is possible that you may become upset while completing the assessment or receiving your parenting program. If this happens, please let the researchers

know, and you are welcome to withdraw from the study at any time. If you get upset or distressed, please seek support from a family member or friend, or call one of the helplines below:

- ***Lifeline: 13 11 14 (24 hours a day, 7 days a week)***
- ***Parentline: 13 22 89 (8am – midnight, 7 days a week)***

2. Although unlikely, there is also a risk to your privacy through breaches of confidentiality, particularly if there is a risk of harm to yourself or others that cannot be prevented without breaching confidentiality. If we believe that yourself, your child, or someone else is at risk of harm (e.g. child abuse, self-harm), we are bound by professional codes of ethics to take reasonable action to prevent this harm occurring, even if it means breaching confidentiality. If this is necessary, we will try to discuss the situation with you (and your child, if appropriate) first.

Reimbursement
You and your teenager will receive a $15 e-gift voucher each after each of the 3-month and 12-month follow-up assessments is completed, to reimburse you for your time. Parents will receive Coles supermarket e-gift vouchers and teenagers can choose between Coles or iTunes vouchers. Vouchers will be sent to you and your teenager separately via email.

What about my privacy?
All information collected will be stored separately from any identifying information, to protect your confidentiality. Information obtained in this research will only be accessible by the researchers named on the project. All electronic files will be password protected. All data will be securely destroyed after a minimum of 5 years from when the final report of the study is published. Any written reports will only include group data, and will not be identifiable in any way.

How can I see the results?
A summary of results will be available in 2018. If you would like a copy, please contact Jacqueline Green at Jacqueline.green@monash.edu. We also aim to have the results published in scientific journals.

What if I have any complaints or concerns?
This project has been approved by the Monash University Human Research Ethics Committee. Should you have any concerns or complaints about the conduct of the project, please contact:

Executive Officer, Monash University Human Research Ethics Committee (MUHREC)
Room 111, Building 3e, Research Office, Monash University, Clayton, VIC, 3800
Tel: +61 3 9905 2052   Email: muhrec@monash.edu   Fax: +61 3 9905 3831

Thank you,

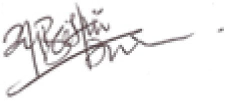

Dr Marie Yap
NHMRC Career Development Fellow
Senior Research Fellow and Psychologist
School of Psychological Sciences
Monash University

YOUTH EXPLANATORY STATEMENT

Partners in Parenting: Evaluating an online personalised parenting program

Chief Investigator: Dr Marie Yap, School of Psychological Sciences, Monash University. Phone: (03) 9905 0723, email: marie.yap@monash.edu
Student Researchers: Jacqueline Green, Doctor of Philosophy candidate; Claire Nicolas, Doctor of Psychology (Clinical) candidate; Peter Martin, Honours student; Brooke Swierzbiolek, Honours student; School of Psychological Sciences, Faculty of Medicine, Nursing and Health Sciences Monash University. Phone: (03) 9905 1250, email: med-partnersinparenting@monash.edu
Senior Research Officer: Shireen Mahtani, School of Psychological Sciences, Monash University. Phone: (03) 9905 1250, email: med-partnersinparenting@monash.edu

Invitation to participate in research

You are invited to take part in research being conducted by Monash University. Please read this Explanatory Statement in full before deciding whether or not to participate. If you would like more information, please contact the researchers via the phone numbers or email addresses listed above.

What does the research involve?
We are interested in how parents can help protect their teenagers from developing problems with depression and anxiety. We want to find out whether a new online program can help parents to do things that might protect their teenager from these problems.

What will I be asked to do?
If you choose to take part, one of the researchers will call you (at a time that suits you) to check that you understand what we are asking you to do, and so that you can ask any questions. We will then ask you to complete an online survey (a researcher will be on the phone to help you do this, if needed). The survey will ask you questions about your parents (e.g. your relationship with them, the things you do together) and also about your feelings and behaviours that may be linked to depression and anxiety (e.g. whether you feel sad or worried about things). The survey will take around 30 minutes.
We will ask you to do a similar survey 3 months later and 1 year later. All of the surveys can be completed whenever you like, as long as you have internet access.
We encourage you to fill out these questionnaires because they let us know whether anything has changed for you and your family.

Who is being asked to take part?
We are asking young people, aged 12 to 15 to be a part of this study, together with one of their parents. You need to live in Australia, speak English, and have access to the internet.

Source of funding
This research project has been funded by Australian Rotary Health.

What if I change my mind?
If, for any reason, you don't want to be a part of this study anymore, you can pull out at any time. No one, not even your parents, can make you continue if you don't want to.

What's in it for me?

There may not be any direct benefit for you taking part in this study, but you will be helping us to find out whether this new online parenting program can reduce depression and anxiety in young people.

When you have completed each of the follow-up surveys 3 months later and one year later, we will email you a $15 iTunes or Coles e-gift voucher to thank you for your time.

Are there any risks to my parent or me?

We don't think there are any major risks of taking part in this study. However, it is possible that you may be upset by some of the questions in the survey. If you do feel upset at any stage, you can call the **Kids' Helpline on 1800 55 1800** to speak to a counsellor. You can call at any time, any day, and there will be someone available to talk to you, for free.

Will other people find out what I say in the surveys?

Everything you tell us as part of this research will be kept private. Even your parents won't be able to find out what you say in the surveys. After you have done the surveys, we will separate your name and other information that can identify you by giving you an ID number. All your information will be password protected.

We will only tell your parents or another adult what you have told us if we think that you or someone else is likely to be hurt. Also, if you become upset while we are on the phone to you, we will ask to speak to our parent to discuss what they can do to support you.

If we think that you may be having some problems with depression and anxiety (based on what you tell us in the surveys), we may suggest to your parents that they take you to a professional who can help you with these problems.

What if I have any complaints or concerns?

This project has been approved by the Monash University Human Research Ethics Committee. Should you have any concerns or complaints about the conduct of the project, you are welcome to contact:

Executive Officer
Monash University Human Research Ethics Committee (MUHREC)
Room 111, Building 3e
Research Office
Monash University, Clayton, VIC, 3800
Tel: +61 3 9905 2052 Email: muhrec@monash.edu Fax: +61 3 9905 3831

Thank you,

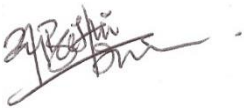

NHMRC Career Development Fellow
Senior Research Fellow and Psychologist
School of Psychological Sciences
Monash University
