# Supplementary material for: A Tailored Web-Based Intervention to Improve Parenting Risk and Protective Factors for Adolescent Depression and Anxiety Problems: Postintervention Findings From a Randomized Controlled Trial
Source: J Med Internet Res. 2018 Jan 19;20(1):e17. doi: 10.2196/jmir.9139 (PMC5797292; doi:10.2196/jmir.9139)
Supplement: Multimedia Appendix 4 [file jmir_v20i1e17_app4.pdf]

Multimedia Appendix 4.

Table A4.1. Observed means and standard deviations of parent and child outcome variables.

Outcome	Intervention<br>M (SD)	Control<br>M (SD)
Parental concordance (PRADAS)		
Baseline	46.2 (7.5)	47.4 (7.7)
Post-intervention	51.6 (8.4)	49.4 (7.5)
Adolescent-reported parental concordance (PRADAS-A)		
Baseline	24.4 (5.6)	24.9 (5.7)
Post-intervention	23.4 (6.3)	24.1 (6.2)
Parent report of child anxiety (Parent SCAS)		
Baseline	18.0 (11.6)	18.5 (12.4)
Post-intervention	15.2 (12.1)	14.8 (11.1)
Child report of anxiety (Child SCAS)		
Baseline	28.7 (17.3)	30.1 (17.4)
Post-intervention	28.3 (20.2)	26.7 (17.1)
Parent report of child depression (Parent SMFQ)		
Baseline	5.0 (5.6)	4.8 (5.0)
Post-intervention	3.6 (4.3)	3.4 (4.4)
Child report of depression (Child SMFQ)		
Baseline	6.2 (6.1)	6.4 (5.8)
Post-intervention	6.2 (6.6)	6.0 (5.5)

Table A4.2. Mixed model repeated measures analyses of primary and secondary outcome scores using log-transformed data.

**Outcome Measure**	**F[a]**	**df**	**p**	d_{post} **(95% CI)**	$d_{interaction}$ **(95% CI)**
SCAS-P	0.04	1, 320	.845	-0.03 (-0.25, 0.19)	0.02 (-0.20, 0.24)
SCAS-C	1.55	1, 306	.213	0.00 (-0.23, 0.22)	0.14 (-0.08, 0.37)
SMFQ-P	0.00	1, 326	.959	0.07 (-0.15, 0.29)	0.00 (-0.22, 0.22)
SMFQ-C	0.00	1, 308	.947	-0.04 (-0.26, 0.19)	0.00 (-0.22, 0.22)

Note. Transformation was not required for PRADAS and PRADAS-A scores, as model residuals for the PRADAS and PRADAS-A MMRMs were normally distributed .

Table A4.3. Moderation effects of parent- and adolescent- reported anxiety and depression scores at baseline on effect of group on parenting, depression and anxiety outcomes.

**Outcome**	**M**	**[a]ΔR^2**	**[b]Moderator level**	**[d]b (95% C.I)**
PRADAS	SMFQ-C	ΔR^2=0.00, $F_{(1,294)=}$0.09, p =.767		
			Low: SMFQ-C≤0.41	3.10 (1.33, 4.87)
			Average: 0.41<SMFQ-C<12.19	2.91 (1.66, 4.16)
			High: SMFQ-C≥12.19	2.72 (0.95, 4.49)
	SCAS-C	ΔR^2=0.01, $F_{(1,294)=}$3.59, p =.0593		
			Low: SCAS-C≤12.42	4.10 (2.35, 5.86)
			Average: 12.42<SCAS-C<46.68	2.90 (1.66, 4.15)
			High: SCAS-C≥46.68	1.71 (-0.48, 3.47)
PRADAS-A	SMFQ-P	ΔR^2=0.01, $F_{(1,303)=}$3.12, p =.078		
			[c]Low: SMFQ-P=0	0.71 (-0.58, 2.00)
			Average: 0<SMFQ-P<9.60	-0.07 (-1.03, 0.88)
			High: SMFQ-P≥9.60	-0.94 (-2.31, 0.42)
	SCAS-P	ΔR^2=0.06, $F_{(1,303)=}$20.09, p =.000		
			Low: SCAS-P≤6.04	2.04 (0.73, 3.36)
			Average: 6.04<SCAS-P<29.30	-0.08 (-1.01, 0.85)
			High: SCAS-P≥29.30	-2.20 (-3.51, 0.88)
SCAS-P	SCAS-C	ΔR^2=0.00, $F_{(1,294)=}$0.01, p =.939		
			Low: SCAS-C≤12.42	0.32 (-2.25, 2.88)
			Average: 12.42<SCAS-C<46.68	0.25 (-1.57, 2.06)
			High: SCAS-C≥46.68	0.18 (-2.39, 2.75)
SCAS-C	SCAS-P	ΔR^2=0.01, $F_{(1,303)}$=2.43, p =.120		
			Low: SCAS-P≤6.04	0.88 (-2.70, 4.45)
			Average: 6.04<SCAS-P<29.30	2.89 (0.36, 5.41)
			High: SCAS-P≥29.30	4.89 (1.32, 8.47)
SMFQ-P	SMFQ-C	ΔR^2=0.02, $F_{(1,294)=}$5.97, p =.015		

			Low: SMFQ-C≤0.41	0.86 (-0.50, 2.21)
			Average: 0.41<SMFQ-C<12.19	-0.33 (-1.29, 0.62)
			High: SMFQ-C≥12.19	-1.52 (-2.88, -0.17)
SMFQ-C	SMFQ-P	ΔR^2=0.00, $F_{(1,303)}$=0.21, p =.651		
			[c]Low: SMFQ-P=0	0.02 (-1.29, 1.34)
			Average: 0<SMFQ-P<9.60	0.23 (-0.74, 1.20)
			High: SMFQ-P≥9.60	0.46 (-0.93, 1.84)

Note. M=Moderator variable. [a]Change in R^2 due to addition of moderator×group interaction term, reported with corresponding F-value and p-value; [b]High and low level values are plus/minus one SD from mean respectively; [c]low values replaced with minimum when one SD below mean placed value outside the range of data; [d]Unstandardised conditional effect of group on outcome scores at corresponding levels of the moderator, reported with 95% confidence interval (CI) in parentheses.
